# Thioredoxin-1 Attenuates Early Graft Loss after Intraportal Islet Transplantation in Mice

**DOI:** 10.1371/journal.pone.0070259

**Published:** 2013-08-09

**Authors:** Kengo Asami, Akiko Inagaki, Takehiro Imura, Satoshi Sekiguchi, Keisei Fujimori, Hiroshi Masutani, Junji Yodoi, Susumu Satomi, Noriaki Ohuchi, Masafumi Goto

**Affiliations:** 1 Division of Advanced Surgical Science and Technology, Tohoku University, Sendai, Japan; 2 New Industry Creation Hatchery Center, Tohoku University, Sendai, Japan; 3 Institute for Virus Research, Biostress Lab, Kyoto University, Kyoto, Japan; 4 Center for Cell Signaling Research/CCSR and Department of Bioinspired Sciences, Ewha Womans University, Seoul, Korea; 5 Division of Surgical Oncology, Tohoku University, Sendai, Japan; St. Vincent's Institute, Australia

## Abstract

**Aims:**

Recent studies suggest that decreasing oxidative stress is crucial to achieve successful islet transplantation. Thioredoxin-1 (TRX), which is a multifunctional redox-active protein, has been reported to suppress oxidative stress. Furthermore, it also has anti-inflammatory and anti-apoptotic effects. In this study, we investigated the effects of TRX on early graft loss after islet transplantation.

**Methods:**

Intraportal islet transplantation was performed for two groups of streptozotocin-induced diabetic mice: a control and a TRX group. In addition, TRX-transgenic (Tg) mice were alternately used as islet donors or recipients.

**Results:**

The changes in blood glucose levels were significantly lower in the TRX group compared with the TRX-Tg donor and control groups (p<0.01). Glucose tolerance and the residual graft mass were considerably better in the TRX group. TRX significantly suppressed the serum levels of interleukin-1β (p<0.05), although neither anti-apoptotic nor anti-chemotactic effects were observed. Notably, no increase in the 8-hydroxy-2′-deoxyguanosine level was observed after islet infusion, irrespective of TRX administration.

**Conclusions:**

The present study demonstrates that overexpression of TRX on the islet grafts is not sufficient to improve engraftment. In contrast, TRX administration to the recipients exerts protective effects on transplanted islet grafts by suppressing the serum levels of interleukin-1β. However, TRX alone appears to be insufficient to completely prevent early graft loss after islet transplantation. We therefore propose that a combination of TRX and other anti-inflammatory treatments represents a promising regimen for improving the efficacy of islet transplantation.

## Introduction

Since the development of the Edmonton protocol, islet transplantation has become an effective option for the clinical treatment of type 1 diabetic patients [1]. However, several donor pancreases are still needed to cure each diabetic patient. In order for islet transplantation to become widespread, the transplantation process must be improved.

The instant blood-mediated inflammatory reaction (IBMIR) that occurs as an innate immune response, characterized by activation of both the coagulation and complement cascades, is a major cause of islet graft loss [2]–[4]. We and others have demonstrated that tissue factor (TF) and monocyte chemoattractant protein-1 (MCP-1) expressed on the grafted islets elicit the IBMIR [4]–[9]. Therefore, to achieve successful outcomes of islet transplantation from a single donor organ, avoidance of the IBMIR is necessary.

On the other hand, pancreatic β cells are also vulnerable to oxidative stress because of their low intrinsic level of antioxidant gene expression [10]. Furthermore, Sklavos et al. reported that redox modulation with catalytic antioxidant protects islets from antigen-independent ischemia-reperfusion injury and hinders the antigen-dependent alloimmune response [11]. Thus, protecting the islet grafts from oxidative stress appears to be another crucial factor to achieve successful islet transplantation.

Thioredoxin-1 (TRX) is a ubiquitously expressed, small (14 kDa) multifunctional protein that has a redox-active disulfide/dithiol within a conserved –Cys-Gly-Pro-Cys- sequence [12]–[14]. TRX protects cells against oxidative stress by scavenging reactive oxygen species, and exerts anti-inflammatory and anti-apoptotic effects by regulating cytokines, signaling molecules and transcription factors [15]–[18]. In addition, TRX regulates neutrophil activation and chemotaxis by acting directly on neutrophils [19]–[21].

In the present study, we investigated the effects of TRX on early graft loss after islet transplantation using a syngeneic mouse model which provides an innate inflammatory milieu without interference from the specific immune system. One of the advantages of islet transplantation, unlike whole organ transplantation, is the ability to modify the grafts during a culture period prior to transplantation. Therefore, if the overexpression of TRX on the isolated islets would improve the outcome of islet transplantation, this can be an ideal and feasible approach with only a limited risk of side effects of TRX. To investigate this possibility, TRX-transgenic (Tg) mice were used as either islet donors or recipients. Furthermore, continuous infusion of exogenous recombinant human TRX (rhTRX) was also performed for the recipients to mimic clinical conditions. Our findings shed light on the potential role of TRX in islet transplantation.

## Methods

### Ethics Statement

All the animals in this study were handled in accordance with the Guide for the Care and Use of Laboratory Animals published by the National Institutes of Health [22]. The protocol was approved by the Committee on the Ethics of Animal Experiments of Tohoku University (Permit Number: 22 IIARE-Animal-24). All surgery was performed under isoflurane (Abbott Japan Co., Ltd., Tokyo, Japan) anesthesia, and all efforts were made to minimize suffering.

### Animals

Male wild type (WT) C57BL/6 mice were purchased from Japan SLR Inc. (Shizuoka, Japan) and male TRX-Tg C57BL/6 mice, in which human TRX complementary deoxyribonucleic acid (DNA) was inserted between the β-actin promoter and its terminator, were raised in our facility. The generation of TRX-Tg mice was described previously [23]. The presence of the human TRX transgene was confirmed by a polymerase chain reaction (PCR) analysis with mouse genomic DNA as a template and synthetic oligonucleotides as primers: forward primer, 5′-CAGATCGAGAGCAAGAC-3′; reverse primer, 5′-CAGGAAACAGCTATGAC-3′. The expression of TRX protein was shown in various tissues in our previous characterization [23].

### Induction and diagnosis of diabetes in the recipients

Diabetes was induced by intravenous injection of 200 mg/kg streptozotocin (SIGMA-ALDRICH, Inc, MO, USA) 5 days before surgery. Mice whose nonfasting blood glucose levels were ≥400 mg/dL on two consecutive measurements were considered diabetic. Serial blood glucose levels were determined, and recipients whose nonfasting blood glucose levels were <200 mg/dL on two consecutive measurements were considered to be cured.

### Islet isolation and transplantation

Before removal of the pancreas, the cannulated bile duct was injected with 4 mL of cold Hanks' balanced salt solution (HBSS) containing 1 g/L collagenase (Sigma type V; Sigma Chemicals, St. Louis, MO, USA). After addition of 4 mL HBSS, the pancreas was digested at 37°C for 12 min. Thereafter, density-gradient centrifugation was performed using Histopaque-1119 (Sigma Diagnostics, St. Louis, MO, USA) and Lymphoprep™ (Nycomed Pharma AS, Oslo, Norway) to isolate the pancreatic islets. The islets were cultured in RPMI-1640 containing 5.5 mmol/L glucose and 10% fetal bovine serum at 37°C in 5% CO_2_ and humidified air before examination.

Diabetic C57BL/6 mice underwent intraportal islet transplantation under isoflurane anesthesia. Murine islets were infused in a total volume of 300 µL into the recipient liver through the portal vein using a 27-gauge Surshield (TERUMO, Inc, Japan).

### Experimental groups

Six islet equivalents (IEQs)/g of syngeneic WT islets were transplanted intraportally into two groups of streptozotocin-induced diabetic WT mice: controls (n = 6) and the TRX (Redox Bio Science, Inc., Kyoto, Japan) -treated group (n = 9). The TRX group was treated with a bolus of rhTRX (40 µg) followed by a continuous infusion (0.4 mg/kg/h) for 7 days. TRX concentration used in this study was in reference to the study of Ueda et al. [20]. Recipients were injected with equivalent amounts of saline as controls. In addition, TRX-Tg islets were transplanted intraportally into streptozotocin-induced diabetic WT mice (TRX-Tg donor group, n = 7), and WT islets were injected into streptozotocin-induced diabetic TRX-Tg mice (TRX-Tg recipient group, n = 7).

### Intraperitoneal glucose tolerance testing

The intraperitoneal glucose tolerance test (IPGTT) was performed 50 days after islet transplantation in the control and TRX groups. D-glucose (2.0 g/kg) was infused intraperitoneally, and the blood glucose concentrations were determined before and at 5, 10, 20, 30, 60, 90 and 120 min after the glucose injection.

### The amount of insulin in the livers of the recipients

Recipient livers were retrieved in 2–7 days after IPGTT and homogenized on ice. After adding deionized water up to 10 mL and 25 mL of 0.18M HCl in 96% ethanol, the homogenate was stored at 4°C for 24 h and was then centrifuged at 2,150 *g* for 10 min. The resulting supernatant was stored at −80°C. The insulin concentration in the supernatant was evaluated using a commercial enzyme-linked immunosorbent assay (ELISA) kit (Mercodia, Uppsala, Sweden).

### Evaluation of islet viability under co-culture with inflammatory cytokines

Freshly isolated islets were cultured with or without 50 U/mL human interleukin (IL)-1β (Roche Diagnostics, Indianapolis, IN), 1000 U/mL mouse tumor necrosis factor (TNF)-α (Roche Diagnostics), and 1000 U/mL mouse interferon (IFN)-γ (Roche Diagnostics) at 37°C for 18 h, then islets were dispersed into individual β cells by treatment with Accutase (Innovative Cell Technologies, San Diego, CA) at 37°C for 10 min. To assess the viability of these cells, the Annexin-V (Becton Dickinson, Franklin Lakes, NJ)/7-Amino-Actinomycin D (7-AAD) assay (n = 5), adenosine diphosphate (ADP)/adenosine triphosphate (ATP) test (n = 3) [24], and ATP/DNA test (n = 3) [25] were conducted.

### Blood analyses

For analyzing the blood samples, the clinically relevant graft dose (8 IEQs/g) was applied. Whole blood samples were collected before and at 6 h after islet infusion, and the samples were clotted for 0.5–1 h at room temperature, then centrifuged for 10 min at 2,200 g. The serum samples were frozen at −80°C. Serum levels of cytokines such IL-1β, IL-6, IFN-γ, MCP-1, and keratinocyte chemoattractant (KC) were determined using the Bio-Plex Suspension Array System (Bio-Rad, Hercules, CA). The serum levels of 8-hydroxy-2′-deoxyguanosine (8-OHdG), a sensitive marker of oxidative stress [26], at 6 h after islet infusion were measured using a Highly Sensitive 8-OHdG Check ELISA (Nikken SEIL Co., Ltd., Shizuoka, Japan). For comparison, we also analyzed the serum levels of 8-OHdG in naive mice.

### Flow cytometric analyses

Hepatic mononuclear cells of recipient mice were prepared as previously described [27]. In this assay, the clinically relevant graft dose (8 IEQs/g) was also applied. Single cells (1.5×10^5^) suspended in phosphate-buffered saline (PBS) with 0.5% bovine serum albumin (BSA) were incubated with saturating concentrations of murine antibodies for 30 min at 4°C on ice in the dark, and subsequently washed and resuspended in PBS with 0.5% BSA. The cell-associated light scatter and fluorescence were determined with the BD FACSCanto™ II instrument (Becton Dickinson). A total of 10,000 viable cells were analyzed. The antibodies used for these analyses were as follows: FITC anti-mouse Ly-6G/Ly-6C (BioLegend, San Diego, CA), APC anti-mouse CD11b (Biolegend), FITC anti-mouse CD3ε (eBioscience, San Diego, CA), and PE anti-mouse αGalCer-CD1d complex (eBioscience). The expression of TF on Gr1^+^ CD11b^+^ cells in recipient livers was detected by a flow cytometric analysis using rabbit anti-mouse tissue factor IgG (American Diagnostica, Stamford, CT), followed by secondary Alexa Fluor 647 donkey anti-rabbit IgG (Invitrogen).

### Histological analyses of transplanted livers

The clinically relevant graft dose (8 IEQs/g) was applied to increase the possibility of finding the grafts in the host livers. The recipient livers with islet grafts were retrieved 24 h after islet infusion and fixed, embedded in paraffin, cut into blocks at regular intervals, and sliced into 4 µm sections. Deparaffinized sections were incubated with a polyclonal guinea pig anti-insulin antibody (Dako, Denmark), then with a goat anti-rabbit EnVision kit (Dako). To detect apoptosis in transplanted islets, TdT-mediated dUTP nick end labeling (TUNEL) staining was performed using a TACS 2TdT-DAB In Situ Apoptosis Detection Kit (Trevigen, Inc.,USA). The number of TUNEL positive islets was counted by double-blind evaluations.

### Statistical analyses

All data are expressed as the means ± SD. Statistical significance was determined using Student's *t* test, the Mann-Whitney U test, or a one- and two-factor analysis of variance with Bonferroni post hoc test. *P* values <0.05 were considered to be significant. An analysis of euglycemic conversion was performed by the Kaplan- Meier method with a log-rank test.

## Results

### The effects of TRX on islet engraftment

**Figure 1:**
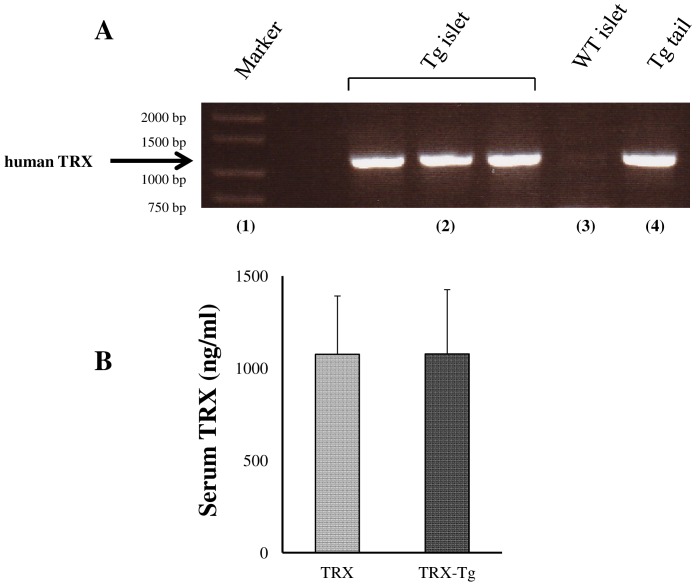
The TRX RNA expression on the islets isolated from TRX-Tg mice. The TRX expression on the islets isolated from TRX-Tg mice was confirmed by PCR (**A**). The TRX band is approximately 1,300 base pairs. (**1**): The size marker (Sigma-Aldrich, Inc), (**2**): Islet DNA from a TRX-Tg mouse, (**3**): Islet DNA from a C57BL/6 mouse, (**4**): Tail tip DNA from a TRX-Tg mouse. **B** The serum levels of TRX at 6 h after islet transplantation in the TRX (n = 5) and TRX-Tg recipient (n = 3) groups. No significant difference was detected between two groups (p = 0.87).

**Figure 2:**
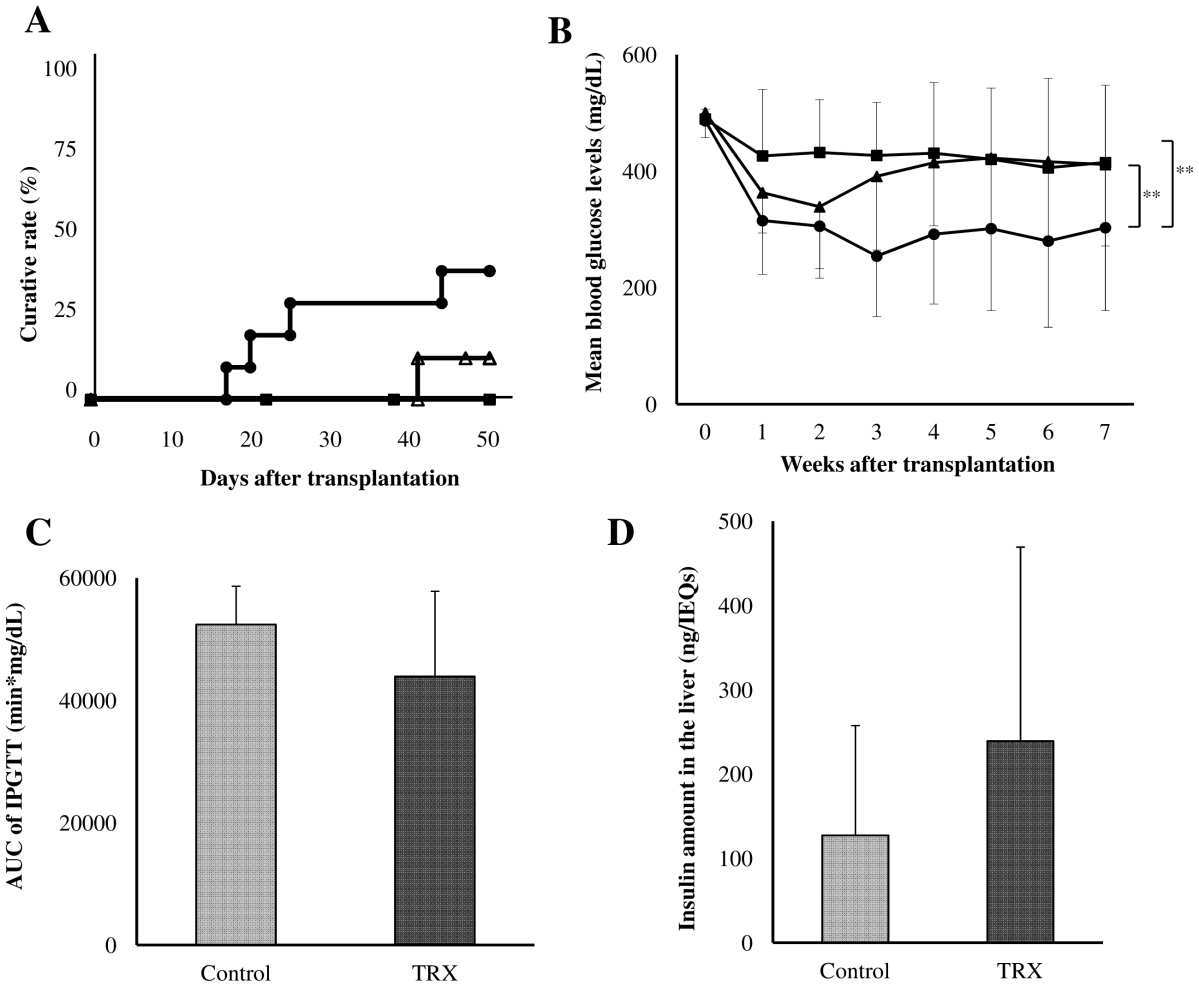
The effects of TRX on islet engraftment. Pancreatic islets isolated from WT or TRX-Tg C57BL/6 mice were transplanted into WT C57BL/6 mice that were rendered diabetic by streptozotocin injection (200 mg/kg intravenously). **A** Diabetic animals received 6 IEQs/g of WT islets with saline (square: Control group), 6 IEQs/g of WT islets with TRX (circle: TRX group), or 6 IEQs/g of TRX-Tg islets (triangle: TRX-Tg donor group). Serial blood glucose levels were measured. Recipients whose nonfasting blood glucose levels were <200 mg/dL on two consecutive measurements were considered to be cured (p = 0.08). **B** The changes in the mean blood glucose levels every week among the three groups were estimated (**p<0.01). **C** IPGTTs were performed 50 days after islet transplantation (n = 3). The results of the IPGTTs were compared using AUC. The glucose tolerance tended to be ameliorated in the TRX group compared to the control group (p = 0.10). Almost all mice in the TRX-Tg donor group were too feeble to undergo the IPGTT. **D** After the IPGTT, the recipient livers in the control and TRX groups were retrieved, and the amount of insulin in the livers of the recipients per transplanted islet was calculated (n = 3). There was a tendency for there to be higher insulin in the TRX group compared with the control group (p = 0.3).

The TRX RNA expression on the islets isolated from TRX-Tg mice was confirmed (Fig. 1A). No significant difference was detected in the serum levels of TRX at 6 h after islet transplantation between the TRX (n = 5) and TRX-Tg recipient group (n = 3, Fig. 1B). All mice used in the present study were severely hyperglycemic (>400 mg/dL) before transplantation. Although none of the 6 mice transplanted with 6 IEQs/g became normoglycemic in the control group, 4 of 9 mice (44.4%) transplanted with the same amount of islets in the TRX group became normoglycemic, and 1 of 7 mice (14.3%) that received the same amount of TRX-Tg islets in the TRX-Tg donor group became normoglycemic during the 7 week study period (Fig. 2A). The mean blood glucose levels were significantly lower in the TRX group compared with the other groups (p<0.01, two-factor analysis of variance, Fig. 2B).

The glucose tolerance showed a tendency to be ameliorated in the TRX group compared to the control group (area under the curve (AUC): 43,901±13953 (n = 5) vs. 52,381±6307 min*mg/dL (n = 3), p = 0.10; the Mann-Whitney U test, Fig. 2C). Furthermore, the amount of insulin in the liver of the recipients also tended to be higher in the TRX group compared with the control group (239.3±230.0 (n = 5) vs. 127.5±130.3 ng/IEQs (n = 3), p = 0.30; the Mann-Whitney U test, Fig. 2D). Almost all of the mice in the TRX-Tg donor group were too feeble to undergo the IPGTT and measurement of insulin in the liver. And all of the 7 diabetic TRX-Tg mice transplanted with WT islets (6 IEQs/g) remained hyperglycemic and died during 9∼45 days (mean 21.7) after transplantation.

### TRX-Tg islets did not protect against injury induced by inflammatory cytokines

**Figure 3:**
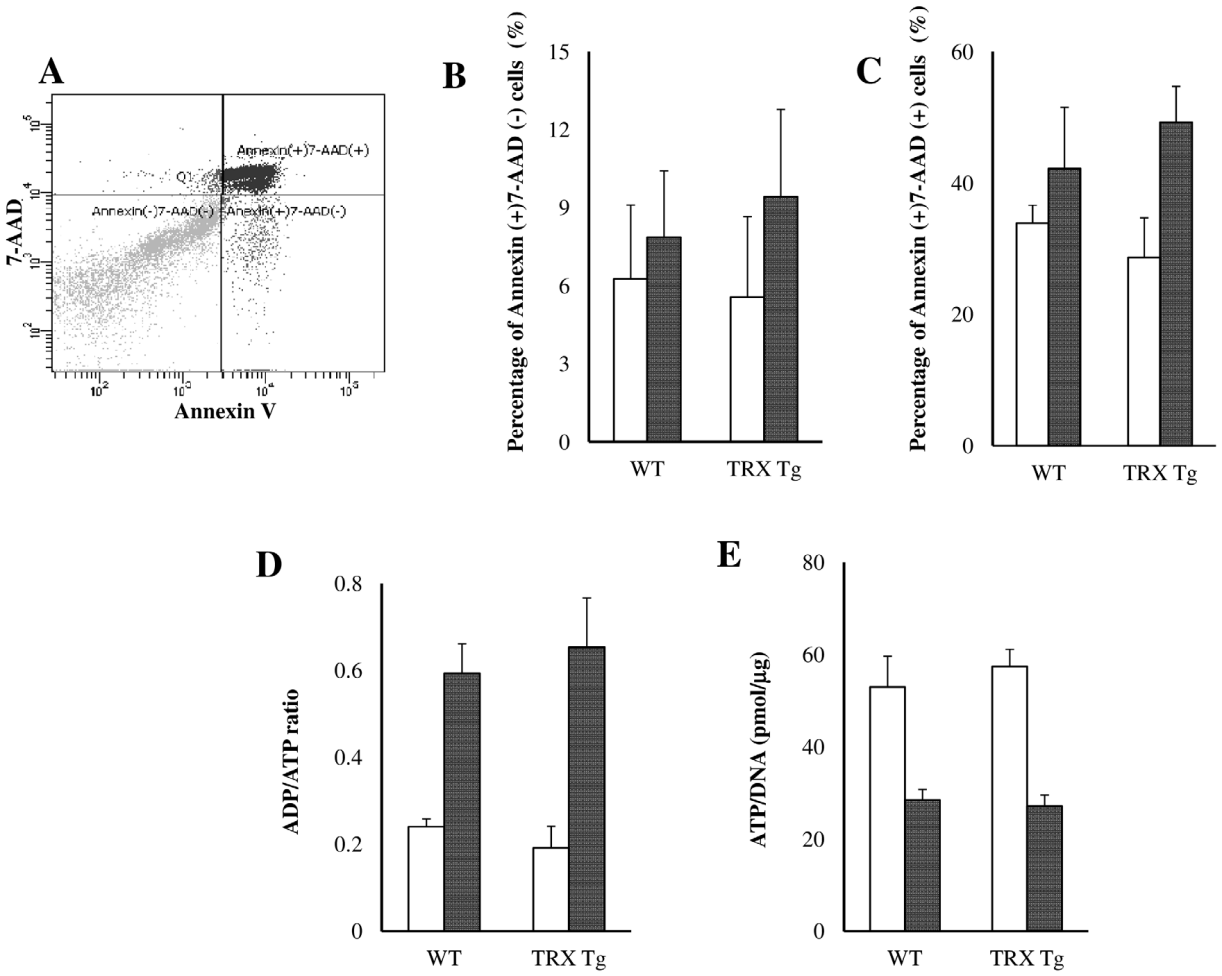
Evaluation of the islet viability under co-culture with inflammatory cytokines. Freshly isolated islets were cultured with (black bar) or without (white bar) human IL-1β, mouse TNF-α, or mouse IFN-γ at 37°C for 18 h, then the islets were dispersed into individual cells by treatment with Accutase at 37°C for 10 min. To assess the viability of these cells, the Annexin-V/7-AAD assay (n = 5, **A, B, C**), ADP/ATP test (n = 3, **D**), and the ATP/DNA test (n = 3, **E**) were conducted.

We evaluated whether TRX-Tg islets had tolerance toward injury induced by inflammatory cytokines in an in vitro assay. In the Annexin-V/7-AAD assay, there were no differences in the percentage of Annexin-V positive, 7-AAD negative cells (namely apoptotic β cells, 9.42±3.35 vs. 7.86±3.10, n = 5; one-factor analysis of variance, Fig. 3A, B), or Annexin-V positive, 7-AAD positive cells (namely dead β cells, 49.26±5.43 vs. 42.20±9.33, n = 5, Fig. 3C) between the TRX-Tg and WT islets co-cultured with inflammatory cytokines. Similarly, no differences were detected between the TRX-Tg and WT islets with regard to the ADP/ATP ratio (0.65±0.11 vs. 0.59±0.07, n = 3; one-factor analysis of variance, Fig. 3D), and ATP/DNA ratio (27.1±2.37 vs. 28.4±2.33, n = 3; one-factor analysis of variance, Fig. 3E).

### The analyses of blood samples

**Figure 4:**
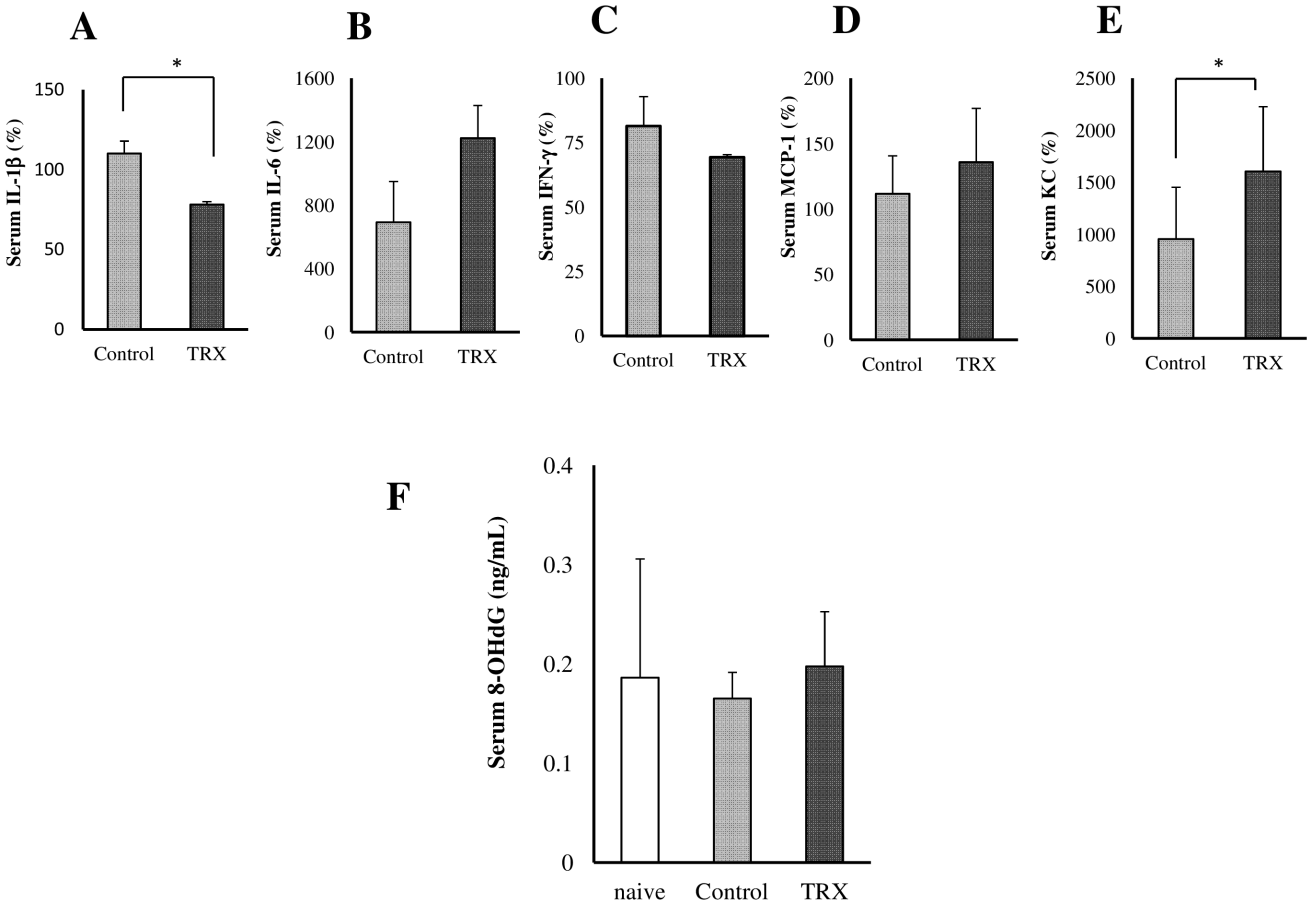
The results of the analyses of blood samples. **A–E** Inflammatory mediators in serum samples were measured before and at 6 h after islet transplantation (8 IEQs/g) (n = 3). All values were expressed as the percentage of the pre-transplant cytokine levels in the serum. Systemic administration of TRX significantly suppressed the serum levels of IL-1β (*p<0.05). In contrast, the serum KC levels in the TRX group were significantly increased compared to the control group (*p<0.05). **F** To evaluate the influence of oxidative stress on transplanted islets, the serum levels of 8-OHdG at 6 h after islet infusion were measured (n = 4). For comparison, we also analyzed the serum levels of 8-OHdG in naive mice (n = 5).

Inflammatory mediators in serum samples were measured before and at 6 h after islet transplantation (n = 3). All values were expressed as the percentage of the pre-transplant cytokine levels in the serum. Systemic administration of TRX significantly suppressed the serum levels of IL-1β (p<0.05, Student's *t* test, Fig. 4A). In contrast, the serum KC levels in the TRX group were significantly increased compared to the control group (p<0.05, Student's *t* test, Fig. 4E). No significant differences in other cytokines such as IL-6, IFN-γ and MCP-1 were observed (Student's *t* test, Figs. 4B–D).

To evaluate the influence of oxidative stress on the transplanted islets, the serum levels of 8-OHdG at 6 h after islet infusion were measured (n = 4). No differences were seen in these groups, but no increase of 8-OHdG was observed after islet infusion in either group (with one-factor analysis of variance, Fig. 4F).

### TRX was unable to regulate the accumulation of Gr1^+^ CD11b^+^, CD1d^+^ CD3ε^+^, and TF positive Gr1^+^ CD11b^+^ cells in the livers of mice receiving islets

**Figure 5:**
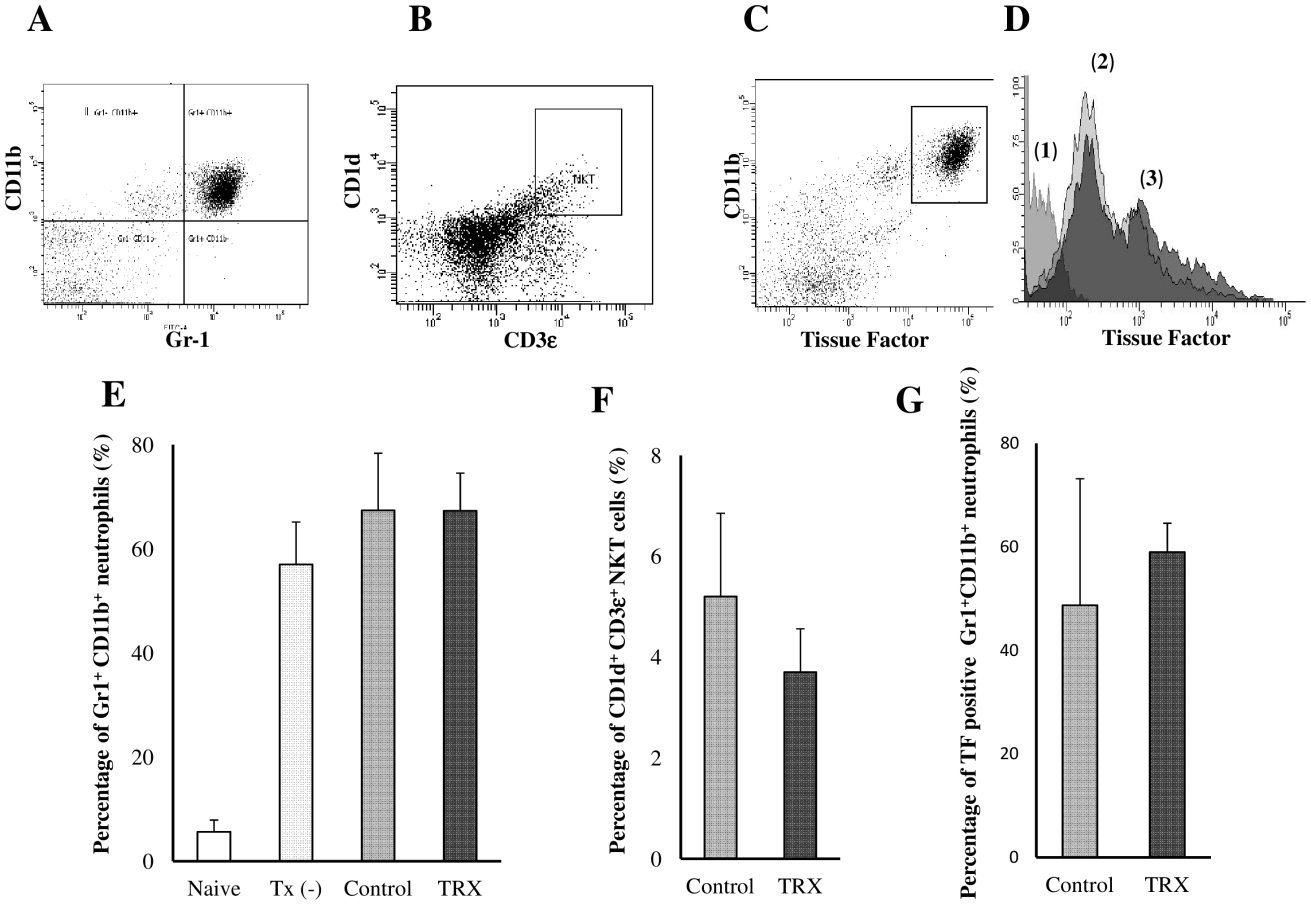
The results of the flow cytometric analyses. We examined whether systemic administration of TRX had any effects on the accumulation of Gr1^+^ CD11b^+^ (n = 3; **A, E**), CD1d^+^ CD3ε^+^ (n = 4; **B, F**), and tissue factor (TF)-positive Gr1^+^ CD11b^+^ (n = 3; **C, D, G**) mononuclear cells in the livers of mice receiving islets in the control and TRX groups. **D** Histogram of TF positive Gr1^+^ CD11b^+^ cells ((**1**); isotype control, (**2**); control, (**3**); TRX). No differences were detected between the TRX and control groups regarding the accumulation of these cells in the transplanted livers.

We examined whether the systemic administration of TRX had any effect on the accumulation of Gr1^+^ CD11b^+^ (mainly neutrophils), CD1d^+^ CD3ε^+^ (natural killer T (NKT) cells), and TF-positive Gr1^+^ CD11b^+^ cells in the livers of mice receiving islets, which are thought to be essential components of early graft loss after islet transplantation [27]–[29]. No differences were detected between the TRX and control groups regarding the accumulation of Gr1^+^ CD11b^+^ cells (Fig. 5E), CD1d^+^ CD3ε^+^ cells (Fig. 5F), or TF- positive Gr1^+^ CD11b^+^ cells (Fig. 5G), with one-factor analysis of variance.

### TUNEL staining of transplanted islets

**Figure 6:**
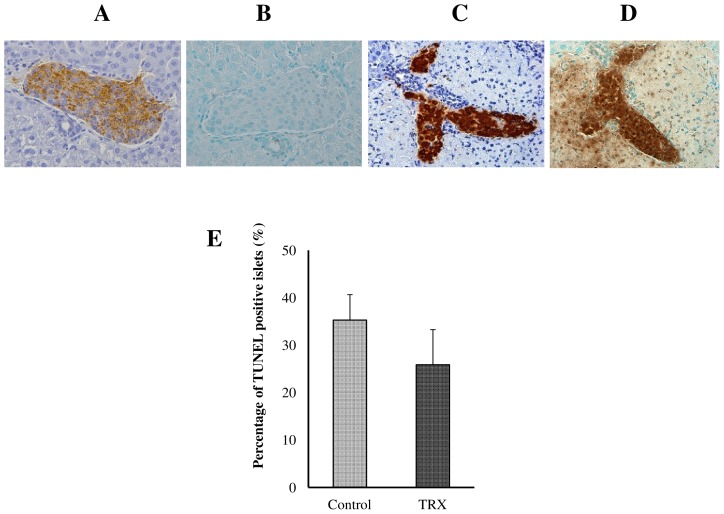
TdT-mediated dUTP nick end labeling (TUNEL) staining of transplanted islets. To examine the anti-apoptotic effect of TRX on transplanted islets, TUNEL staining was performed for the transplanted livers at 24 h after islet transplantation in the TRX and control groups (n = 3). The same islets were stained with both insulin (**A, C**) and TUNEL (**B, D**), respectively. **B** A TUNEL negative islet, **D** A TUNEL positive islet., **E** The rate of TUNEL positive islets was lower in the TRX group than in the control group, but the difference did not reach statistical significance (p = 0.28).

To confirm the anti-apoptotic effects of TRX on transplanted islets, TUNEL staining was performed for the transplanted livers at 24 h after islet transplantation in the TRX and control groups (n = 3). The rate of TUNEL positive islets was lower in the TRX group compared with the control group, but the difference did not reach significance (25.9±7.42 vs. 35.3±5.37, p = 0.28; the Mann-Whitney U test, Fig. 6E).

## Discussion

Several recent studies suggested that oxidative stress, as well as the IBMIR, appears to be one of the crucial factors affecting successful islet transplantation [30], [31]. TRX has been reported to effectively suppress oxidative stress [32]. Moreover, it also exhibited anti-inflammatory and anti-apoptotic effects in various experimental models [15]–[18]. Therefore, we examined the effects of TRX on early graft loss after islet transplantation.

Unexpectedly, the TRX locally expressed on islet grafts was insufficient to protect them from the innate immune system, although systemic administration of TRX to the recipients appeared to be effective. Our present *in vivo* data were also supported by the *in vitro* study using TRX-Tg islets and the assessment of inflammatory cytokines including IL-1β, TNF-α, and IFN-γ (Fig. 3). These findings suggest that a substantial amount of TRX is required to protect the grafts after islet transplantation. On the other hand, Chou FC et al. previously reported that overexpression of TRX did prolong graft survival against inflammatory insults [32]. One possible explanation for this discrepancy is that the expression level of TRX by lentivirus in their study may be far higher than that of the TRX-Tg mice. The dosage effect of the localized overexpression of TRX should be further examined.

In the present study, we found that the serum levels of IL-1β were significantly suppressed in the TRX group. Corroborating our findings, Yoshioka et al. and Billiet et al. have reported that TRX could regulate the activation of NF-κB, followed by the suppression of IL-1β production [17], [33]. It has been reported that pancreatic islets are highly sensitive to proinflammatory cytokines such as IL-1β [34]–[36]. Montolio et al. [37] reported that IL-1β mRNA in the transplanted islets was upregulated immediately after islet infusion, and that IL-1β played a crucial role in the extensive β-cell death found in the initial days after islet transplantation. Taken together, the suppression of IL-1β observed in the present study may contribute to the protection of the transplanted islet grafts by TRX.

In contrast, the serum level of KC, the murine homologue of human IL-8, was increased in the TRX group compared with the control group. Consistent with this finding, Bertini et al. [38] reported that TRX *per se* was chemotactic for monocytes, polymorphonuclear leukocytes, and T lymphocytes. This may partially explain why no beneficial effects of TRX were seen in some of the TRX-treated mice. In view of its potential clinical applications, further investigations of the independent effects of KC on islet engraftment are needed.

The serum levels of 8-OHdG were measured to assess the involvement of oxidative stress in the transplanted islets. No increase in the 8-OHdG levels was observed after islet infusion even in the control group, suggesting that oxidative stress, at least in this model, has a minimal effect on islet transplantation. These results imply that the upregulated inflammatory mediators, rather than oxidative stress, may have stronger impact on the outcome of islet transplantation.

To determine the significance of the systemic distribution of TRX in the recipients, streptozotocin-induced diabetic TRX-Tg mice were also used as the recipients. Notably, the TRX-Tg mice were especially sensitive to streptozotocin, and this toxicity became pronounced following surgical stress, irrespective of the islet infusion, and resulted in both liver and renal damage, though it was previously reported that histological analysis of the pancreas revealed similar extent of beta cell destruction between wild type and TRX-Tg mice after streptozotocin treatment [39]. The detailed mechanism underlying this effect remains uncertain, but likely reflects the multifunctional roles of TRX. Subsequent analyses using this model were not performed in the present study.

In the FACS analysis, unlike previous reports in which lipopolysaccharide (LPS)-induced neutrophil infiltration was effectively suppressed by rhTRX [19], [20], we were unable to confirm the role of TRX in the regulation of neutrophil chemotaxis to the liver at 6 h after islet transplantation. One possible explanation for this discrepancy is the cause of the neutrophil infiltration; TRX may inhibit the specific signaling pathway activated by LPS. Of particular interest, the accumulation of Gr1^+^ CD11b^+^ cells in the liver was significantly increased by infusion of transplant medium itself (without any islets) to the liver. This indicates that neutrophil chemotaxis after intraportal islet transplantation can be attributed to not only islet grafts, but also to the transplant procedure itself. In this FACS analysis, we also evaluated the TF expression on leukocytes in the liver, since NF-κB suppression is known to cause downregulation of TF [40], [41]. However, the accumulation of TF-positive neutrophils was not affected by TRX, suggesting that TRX is not related to the inhibition of the IBMIR.

One of the strong points of TRX treatment is its safety. TRX is expected to be easily approved for testing in clinical trials, since it is currently in a phase II clinical trial for the treatment of acute respiratory distress syndrome.

In summary, the present study demonstrates that localized overexpression of TRX on the islet grafts is not sufficient to improve their engraftment. In contrast, exogenous TRX administration to the recipients exerts protective effects on transplanted islet grafts by suppressing the serum levels of IL-1β. However, TRX alone appears to be insufficient to completely prevent early graft loss after islet transplantation. We therefore propose that the combination of TRX and an anti-IBMIR treatment, such as C5a inhibitory peptide [27], represents a promising regimen for improving the efficacy of islet transplantation, although further optimization will be required in a clinical setting.
